# Do different spectral domain OCT hardwares measure the same? Comparison of retinal thickness using third-party software

**DOI:** 10.1007/s00417-015-3075-2

**Published:** 2015-06-12

**Authors:** Birgit Sander, Hajer Ahmad Al-Abiji, Mads Kofod, Thomas Martini Jørgensen

**Affiliations:** Department of Ophthalmology, Rigshospitalet - Glostrup, Ndr. Ringvej 57, DK-2600 Glostrup, Denmark; Department of Applied Mathematics and Computer Science, Technical University of Denmark, Richard Petersens Plads, Building 324, DK-2800 Kgs. Lyngby, Denmark

**Keywords:** OCT, Spectral domain, Retinal thickness, Outer segments, Segmentation

## Abstract

**Purpose:**

Spectral-domain optical coherence tomographies (OCTs) from different companies do not give identical retinal thicknesses. The purpose of this study was to evaluate if differences in thickness when using a spectral domain Cirrus OCT or a Heidelberg Spectralis are due to hardware differences, or if they are caused by the segmentation algorithms.

**Methods:**

Thirty-seven healthy eyes were examined within the same session with a Cirrus OCT and a Spectralis OCT, the latter using averaged B-scans. Scans from similar positions and passing the fovea were analyzed by custom-made software. Thickness was analyzed at the fovea, the central 1-mm line and the 6-mm line.

**Results:**

When Cirrus and Spectralis scans were analyzed with the same software, the retinal thickness at the foveal center was 225.92 μm (SD 17.0) using the Cirrus and 228.70 μm (SD 18.4) using the Spectralis; the difference of 2.78 μm was not significant (*p* = 0.055). For the central 1 mm, the difference was 1.78 μm (*p* = 0.0414), and for all points out to 6 mm, the Spectralis retinal thickness was also significantly larger than the Cirrus thickness (*p* = 0.0052), though the mean difference was only 1.85 μm. Also for the RPE_OS_complex_, Spectralis measured a greater thickness than did Cirrus, with a mean of 3.32 μm (*p* < 0.0001) for all points.

**Conclusion:**

The retinal thicknesses from the Cirrus and from the Spectralis differed by 14 μm with the standard software of the instruments, and by less than 3 μm when analyzed with the same custom-made software, indicating that the major differences between the two SD-OCT systems are due to differences in their built-in software algorithms.

## Introduction

Optical coherence tomography (OCT) is used for the quantification of retinal thickness in a large number of retinal diseases, and the present technology is based on a spectral domain detection system, illumination at 840 to 870 nm and scanning speeds in the range of 20–50,000 A-scans/sec. The resolution of ophthalmic OCT is typically around 5–7 μm in the axial direction and 10 μm in the lateral dimension [1, 2]. The measurement of retinal thickness is considered a reliable and reproducible measurement in healthy subjects [3], and is used in daily clinics for a large number of retinal diseases with severe pathology, with some decrease in reproducibility [4].

Many OCT devices are available and the instruments often differ in the software algorithms used for segmentation, leading to considerable differences in the nominal retinal thickness [1–7]. The inner border is chosen uniformly to the vitreoretinal surface, which is well defined with an abrupt change in reflectance for the nearly optically empty vitreous body to the reflecting inner limiting membrane and retinal nerve fiber layer surface of the retina. The discrepancies between the inbuilt software systems are particularly due to different definitions of the outer retinal border, which is defined anywhere between the junction between inner and outer segments to the posterior part of the retinal pigment epithelium and Bruch’s membrane complex surface.

In previous time domain OCTs, the Stratus OCT used the junction between inner and outer segments as the outer border. This definition was probably chosen because this line is the first highly reflecting line in the neuroretina, apart from the vitreoretinal surface. The segmentation is not ideal as a measurement of retinal thickness, as the outer segments, and therefore a part of the neuroretina, were excluded. For spectral domain instruments, the definitions have changed, and Cirrus HD OCT defines the outer border at the level of interdigitation between outer segments and the retinal pigment epithelium (RPE) following the anatomical concept of the outer border of the neuroretina [4, 8]. In an OCT image, the junction is represented by a second hyper-reflective band beneath the junction of the inner and outer segments, and this second line is most clearly visible in the foveal area. The Heidelberg Spectralis system has chosen the posterior part of the third hyper-reflective band from the OCT, corresponding to the level of the RPE—Bruch’s membrane complex (RPE_outer_) [4, 9]. An advantage of this definition is the clear distinction of the underlying choriocapillaris layer, which appears less reflective, partly due to absorption in the overlying RPE. The measured retinal thicknesses for healthy subjects are therefore dependent on the instrument and the applied definitions for segmentation. Using the program 3D-OCTOR, Heussen et al. [10] compared the thickness for the foveal retinal thickness (FRT) with a manual segmentation and found only small differences between SD instruments. The outer border in the study was the interdigitation of the outer segments and the RPE; however, this line is located between two highly reflecting lines (IS/OS and RPE) and is more difficult to visualize, in particular outside the fovea.

The posterior border is a high contrast border and defines the outermost part of the retinal epithelium—Bruch’s membrane complex (RPE_outer_). The objective of the present study is to compare retinal thicknesses of the Cirrus and Heidelberg Spectralis OCTs using the RPE_outer_ both for the foveal center and perifoveal points, using a semi-automatic segmentation method. In addition, the thickness of the RPE_OS_complex_ has been analyzed.

The small thicknesses of the outer segments are not calculated in any of the standard software accompanying the hardware. For a robust measurement of the outer retina, the new software also measures the distance from the junction of inner and outer segments to the RPE_outer_, termed the RPE_OS_complex_, including the outer photoreceptor segments as a major part. The RPE_OS_complex_ has previously been shown to be predictive of visual acuity after macular hole surgery [11].

## Materials and methods

### Subjects

Thirty-seven eyes in 37 patients were included in the study. The patients were a subset of patients enrolled in a clinical study of epiretinal membrane aiming to determine the effect of early surgery on this condition, where the innermost part of the inner limiting membrane is thickened (ClinicalTrials.gov: NCT00902629). The original study included 113 patients, and no pathology was apparent in 64 eyes [12]. The clinical examination included visual acuity measured with standardized early treatment diabetic retinopathy study (ETDRS) charts, ophthalmological examination and OCT. The decision of healthy conditions was based on a clinical evaluation of the patient by one of the authors (MK) and the absence of subjective visual complaints.

### OCT procedure

For the present analysis, the selection was based on the presence of high quality scans from both the Cirrus OCT (Carl Zeiss Meditec Inc., Dublin, CA) and Heidelberg Spectralis (Heidelberg Engineering Inc., Heidelberg, Germany) from the same visit and with comparable positions. The position and centering of the scans are critical for the calculated thicknesses, and all scans were evaluated by two of the authors (HAA,BS) and excluded in case of different positions. Accordingly, for all eyes included, the scans were centered on the fovea with absence of inner retinal layers. A total of 54 patients were available with scans recorded at the same day on both OCT machines; 16 were excluded due to differences in position. The recommended threshold for signal quality is a signal strength of 6 on the Cirrus and a quality of 15 on the Heidelberg instrument, and one eye was excluded due to low signal strength on the Cirrus and poor definition of the fovea, leaving 37 eyes for the study [13].

The scanning protocol for the Cirrus OCT consisted of the standard five-line protocol, with a nominal scanning length of 6 mm (512 A-scans) without averaging, while the protocol for the Heidelberg Spectralis was a horizontal line of 30 degrees (768 A-scans, 8.7 mm) and the averaging of 100 raw B-scans. The individual scanning length from each Heidelberg examination was obtained from the image header information. All scans were performed by trained staff (MK and HAA) after dilatation.

### Segmentation

Custom image segmenting software and quantification were implemented to ensure that the retinal thicknesses were calculated in the same manner for both the Cirrus and Heidelberg. The segmentation lines were automatically obtained using a so-called shortest path algorithm based on dynamic programming, as described in [14, 15].

Initially, the B-scan is aligned horizontally by maximizing the intensity correlation between neighboring A-scans. A rough estimate of the location of the center of the RPE layer is obtained by picking the image row with the highest intensity sum, since the RPE layer is hyper-reflective. Next an improved estimate of the center of the RPE is obtained by calculating the “shortest path” in the negated B-scan (i.e. the hyper-reflective areas become dark) in a narrow vertical region around the centerline. The length of the path is given by the sum of the pixel values along the path. The top of the IS/OS layer is found by calculating the “shortest path” across the negated vertical gradient image obtained from the initial B-scan, and by searching above the calculated centerline. The bottom of the RPE layer is found by calculating the “shortest path” across the negated vertical gradient image obtained from the initial B-scan, and by searching below the calculated centerline. The innermost border of the retina towards the vitreous is found by searching a negated vertical gradient image of the OCT scan above the detected IS/OS RPE layer.

In a few cases, the automated segmentation would produce some erroneous segmentation. In these cases, the user was allowed to click on specific points of the “true” segmentation curves to guide the shortest path algorithm.

**Fig. 1:**
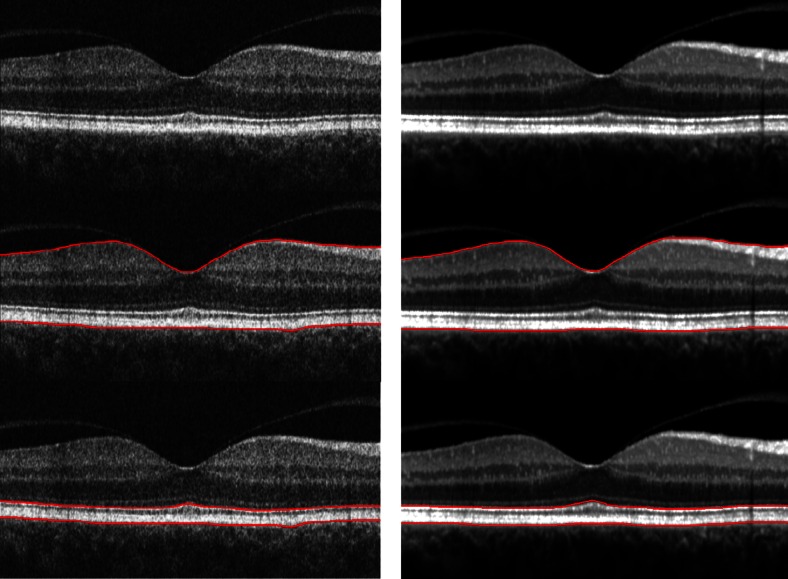
Illustration of the segmentation lines of the third-party software. The *left panel* is based on Cirrus, the *right panel* on Heidelberg Spectralis. *Top image*: 6-mm horisontal OCT scan from of a healthy eye. The posterior hyaloid is detached both temporal and nasally to the fovea; this does not disturb the segmentation algorithm. *Middle*: The segmentation of the inner retina (the vitreo-retinal surface) and the outer retina (the RPE_outer_); these lines are used for calculation of the retinal thickness. *Bottom*: For the thickness of the outer retina (the RPE_OS_complex_), the innermost line is defined at the level of the inner and outer segment junction (IS/OS) and the outer line is unchanged. As the Heidelberg Spectralis is based on averaged images, the contrast is higher

The automated segmentation lines were calculated by the software and were thereafter manually inspected. Misalignments were corrected by the interactive procedure. Two thickness measurements were evaluated: the retinal thickness, defined as the distance from the innermost border of the retina towards the vitreous, as is the standard with all OCT software, and for the outer border, the RPE_outer_, i.e. the retinal pigment epithelium—Bruch’s membrane complex was chosen, due to the marked transition in reflectance at this border (Fig. 1). As a second outcome, the thickness of the RPE_OS_complex_ was calculated. This layer includes the retinal pigment epithelium, the relatively low reflecting transition zone to the outer segments, the outer segments and the inner segmentation line at the interdigitation of outer and inner segments. This line seems to reflect the ellipsoid of the photoreceptor cells, with a large number of mitochondria assumed to be the origin of the high reflectance of this layer [16].

Retinal thickness was measured in the foveal center (central point thickness, CPT) and for the central 1-mm line crossing the fovea with a 0.5 mm distance to both the nasal and the temporal side, and for all points up to 3 mm from the fovea, i.e., a total nominal diameter of 6 mm. For the Cirrus OCT, the 3-mm point was not always available both nasally and temporally, as this needs perfect centering of the scan on the fovea. In total, 31 points were missing, approximately half on each side.

For comparison to the built-in software calculations, the central subfield (CSF), i.e., the mean thickness of the central circle that is 1 mm in diameter, was obtained from both instruments using the relevant mapping protocol. With the Cirrus, the mapping protocol was a 6-mm map with 128 raster lines, each with 512 A-scans; and with the Heidelberg Spectralis, it was a 20-degree map, (512 A-scans, 6.2*6.2 mm) 49 B-scans, each based on ten averages. In addition, the central point thickness was obtained from the Spectralis software.

For the patients included in the study, repeated measurements were available for a subset of patients (*n* = 12, time between measurements = 3 months), and these data were analyzed to evaluate repeatability.

The study was approved by the regional ethical committee of the capital region of Copenhagen, Denmark, and informed consent was obtained for all patients.

### Statistical analysis

The thickness data were summarized as means and standard deviations (SD). Paired *t* tests were used for comparison of retinal and outer layer thickness of the different OCT equipment and software programs. Data were analyzed for the foveal center, the 1-mm central line, using the points 0.5 mm nasal to fovea, foveal center and 0.5 mm temporal (corresponding to the 1-mm wide CSF area). Also, data was analyzed for all points for the 6-mm line, from 3 mm nasal to 3 mm temporal. Due to the paired analysis, data for the 3-mm point on Heidelberg were omitted from tables and calculations for the 31 points where Cirrus data was not available. The analysis of all points on the 6-mm line was adjusted for multiple measurements using a mixed model, with the differences between the Cirrus and Heidelberg as dependent variables and a repeat statement for the measurement points along the 6-mm line. For correlations, Pearson’s *r* value was applied and a regression model was used to calculate the transformation of Cirrus data to the definition used by the custom-made software. Bland-Altman plots were included in the analysis.

For repeatability (intra-device), the mean absolute difference was calculated, as was the Bland-Altman coefficient of repeatability (expressed in % of the mean thickness). Calculations were performed using SAS statistical software (SAS version 9.3., SAS Institute Inc., Cary, NC, U.S.A.). A *p* value < 0.05 was considered significant.

## Results

Thirty-seven patients were included in the study, with a mean age of 67.74 years (range 54 to 77); 15 were male and 22 females. The mean visual acuity was 86.59 ETDRS letter (range 77 to 96), which is equivalent to a Snellen acuity of 20/20. The central subfield thickness (CSF) with the inbuilt standard software was 269.83 μm (SD 21.5) for the Cirrus and 284.08 μm (20.45) for the Heidelberg Spectralis. This difference of 14.25 μm was significantly different (*p* < 0.0001) as expected due to the different definition of the standard software.

Initially, our software algorithm was compared to the standard software available from the Heidelberg Spectralis, as the definitions of segmenting layers are similar. The foveal center point is the minimal thickness of the line scan, and was found to be 228.01 μm (SD 17.47) when obtained from the standard software, and to be 228.70 μm (SD 18.42) from custom-made software. The difference was not significant (*p* = 0.17) and the correlation was linear with a Pearson correlation coefficient of 0.99 (*p* < 0.0001).

Foveal center: Using the same third-party software for both instruments, the foveal point thickness was calculated to be 225.92 μm (17.02) for the Cirrus and 228.70 μm (SD18.42) for the Heidelberg. The difference was just outside significance at the 5 % level (*p* = 0.055, Table 1), and the correlation was linear with Pearson’s *r* = 0.85 (*p* < 0.0001). For the central point thickness of the RPE_OS_complex_, a significant difference was found for the foveal point, as the thickness was 76.05 μm (SD 6.11) with the Cirrus and 78.49 μm (SD 5.00) with the Heidelberg (*p* = 0.0136, see Table 2). The correlation between the Cirrus and the Heidelberg for the RPE_OS_complex_ was 0.52 (*p* = 0.0021).

**Table 1:** Retinal thickness as measured with third-party software on OCT scans from the Cirrus HD and Heidelberg Spectralis systems. Results are reported using means and SDs for the foveal center (top), the central 1-mm line crossing the fovea (middle), and all points of the 6-mm line (bottom). The difference between the instruments is given in μm, and the percentage and the *p* value are also for comparison of the instruments

Retinal Thickness	Cirrus HD	Heidelberg Spectralis	Difference	% Difference	*p* value
	μm	μm	μm		
Foveal center, mean	225.92	228.70	2.78	1.22	0.0555
SD	17.02	18.42	8.56		
1-mm line, mean	280.37	282.15	1.78	0.63	0.0414
SD	17.90	18.40	5.13		
All points, mean	301.10	302.95	1.85	0.62	0.0052
SD	39.07	38.57	7.50

**Table 2:** The thickness, as measured with third-party software on OCT- scans from Cirrus HD and Heidelbarg Spectralis systems. Results are reported using means and SDs for the foveal center (top), the central 1-mm line crossing the fovea (middle), and all points of the 6-mm line (bottom). The difference between the instruments is given in μm, and the percentage and the *p* value are also for comparison of the instruments

RPE_OS_complex_	Cirrus HD	Heidelberg Spectralis	Difference	% Difference	*p* value
	μm		μm	%	
Foveal center, mean	76.05	78.49	2.43	3.14	0.0136
SD	6.11	5.00	5.70		
1-mm line, mean	68.52	71.77	3.25	4.63	< 0.0001
SD	3.86	3.78	3.11		
All points, mean	61.59	64.91	3.32	5.29	< 0.0001
SD	6.71	6.35	4.40		

Central 1 mm: When data were analyzed for the 1-mm central part of the scan, the thickness from the Heidelberg OCT was 1.78 um larger than that from the Cirrus HD OCT, and the difference was significant (*p* = 0.0414). For the RPE_OS_complex_, the difference was 3.25, and again that of the Heidelberg Spectralis was thicker than that of the Cirrus, and the difference was significant (*p* < 0.0001).

**Fig. 2:**
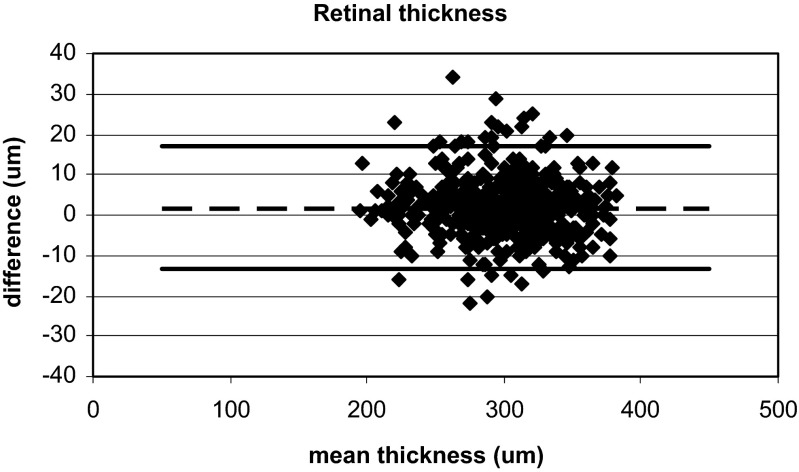
Bland-Altman plot of the retinal thickness for all measurements points with the Cirrus OCT and the Heidelberg OCT. The y-axis shows the difference in retinal thickness (μm) between measurements obtained using the Heidelberg OCT and Cirrus OCT, and the x-axis shows the mean sum of the two instruments. The mean difference of 1.85 μm is shown with a *stippled line*, and the mean ± 2SD is shown with *solid lines*

**Fig. 3:**
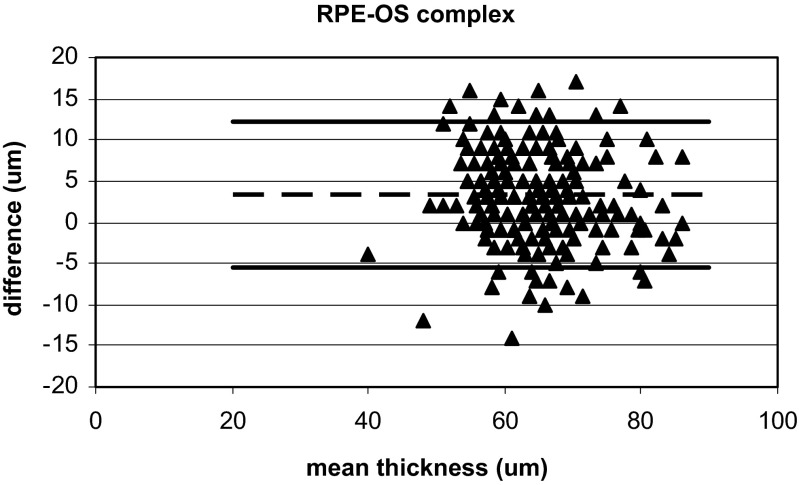
Bland-Altman plot of the outer retinal thickness, the RPE_OS_complex,_ for all measurement points with the Cirrus OCT and the Heidelberg OCT. The y-axis shows the difference in thickness (μm) between measurements obtained using the Heidelberg OCT and Cirrus OCT, and the x-axis shows the mean sum of the two instruments. The mean difference of 3.32 μm is shown with a *stippled line*, and the mean ± 2SD is shown with *solid lines*

All points: Calculated for all points of the 6-mm scan, the mean difference in retinal thickness was 1.85 μm (*p* < 0.0052) and for the RPE_OS_complex_, the difference was 3.32 mm (*p* < 0.0001). No effect was found for age or gender (*p* > 0.5). When analysed by a Bland-Altmann plot (Figs. 2 and 3), no particular pattern was found for retinal thickness or the RPE_OS_complex_.

The signal strength is calculated differently in the instruments; for the Cirrus, mean signal strength was 8.08 (range 6 to 10), while for the Heidelberg Spectralis, the signal to quality index was 34.36 dB (range 26 to 41). Analyzed by spearman correlation, the correlation coefficient was 0.01 and clearly non-significant (*p* > 0.9).

For conversion of Cirrus standard retinal thickness to Heidelberg standard thickness with a definition using RPE_outer_ as the posterior border, the conversion was 28.945 + Cirrus thickness X 0.9482 (CSF: 1 mm area). For the conversion of the third-party software, the conversion factor from Cirrus to Heidelberg was 5.2852 + Cirrus thickness X 0.9875 (1 mm horizontal line).

The intra-device variability was calculated as the mean difference in thickness for a subset of patients (*n* = 12). For retinal thickness, the mean difference for all points of the 6 mm line was 4.73 μm (SD 4.54) for the Cirrus and 4.80 μm (SD 3.84) for the Heidelberg. The corresponding values for the central 1-mm line was 3.23 um (SD 1.79) for the Cirrus and 3.63 μm (SD 3.06) for the Heidelberg, and the Bland-Altman coefficient of repeatability was 3.27 % for the Cirrus and 4.02 % for the Heidelberg. For the RPE_OS_complex_, the intra-device variability was 3.38 μm (SD 2.97) for the Cirrus and 2.63 μm (SD 2.64) for the Heidelberg for all points. The corresponding values for the 1-mm line was 1.78 μm (SD 1.43) for the Cirrus and 1.64 μm (SD 1.57) for the Heidelberg, and the Bland-Altman coefficient of repeatability was 6.67 % for the Cirrus and 6.33 % for the Heidelberg

## Discussion

OCT is used for diagnosis and follow-up in many retinal diseases, and often the central thickness is used as a guideline for treatment decisions; it is used extensively in neovasular age-related macular degeneration [17]. Clinics have different OCT equipment, which may lead to differences in retinal thickness, and even for SD instruments, the definitions of the posterior border of the retina is not the same. In the present study, we analyzed the retinal thickness, defined as the distance from the vitreoretinal border to the RPE_outer_ and the thickness of the RPE_OS_complex_, using the same software algorithms on OCT scans from Cirrus HD OCT and Heidelberg Spectralis OCT.

An initial calculation showed that the central point thickness from our custom-made software was comparable to that reported earlier, with a nonsignificant difference of 0.69 um compared to a direct read out of the standard Spectralis software [9]. A small difference of 3.22 um was also found by Lee et al., where the Spectralis software was 3.22 μm thicker compared to a another automatic software, with a high intra-class coefficient (ICC) of 1.0 [18].

When comparing the Cirrus and Spectralis instruments with our custom-made software, the center point thickness was 225.92 μm from the Cirrus and 228.70 μm from the Heidelberg Spectralis, and the mean difference of 2.78 μm was just outside 5 % significance (*p* = 0.0555). Thus the third-party software used here gives identical results to the Heidelberg Spectralis standard program for the Heidelberg OCT, and when applied on the Cirrus scans, the central point was slightly thinner, by less than 3 μm.

For the RPE_OS_complex_, the differences were larger. For the foveal center, the difference was 2.43 μm (*p* = 0.0136), and for all measurements, the difference was 3.32 μm (*p* < 0.0001).

For the thickness of the central 1-mm horizontal line, we also found slightly larger values using the same software for the Heidelberg Spectralis compared to the Cirrus OCT, with a mean difference of 1.78 μm (*p* = 0.0414). Heussen et al. [10] compared Cirrus HD OCT to Heidelberg Spectralis for the foveal central subfield area using the 3D-OCTOR program with manual segmentation. The outer border in this study was the interdigitation of the outer segments and the RPE, comparable to the definition used in the Cirrus standard software. Heussen found a difference of 3.7 μm, slightly above the 1.78 found in this study; in contrast to our study, the difference was not significant. The line of interdigitation used by Heussen et al. is weak, in particular outside the fovea, thus for the present study, we used a more robust definition, applicable over the entire length. Rashid et al. reported a mean difference of 6.76 μm (Heidelberg Spectralis thinner) for the retinal thickness in 11 eyes, including four healthy eyes [19]. The number of eyes is small, and the difference is opposite to that of our results, underlining that the small differences found in both our and other studies should be interpreted with caution. The repeatability coefficient of our study was below 5 % for the measurement of retinal thickness and below 10 % for the RPE_OS_complex_, and thus comparable to earlier studies with the same two instruments [20, 21].

For all points of the 6-mm scan, the mean difference was 1.85 μm. Though the difference was significant (*p* < 0.0052), it was below 1 % of the mean retinal thickness and well below the intra-device variation, and not clinically relevant. The result indicates that the difference between Cirrus and Heidelberg results is primarily due to the software algorithms and definitions of segmentation, and that other instrument features are minor. When these differences are corrected by using the same segmentation algorithms on both devices, the remaining differences are small, as shown by the present study. Different SD-OCT devices typically have different contrasts between high and low intensity signal, different pixel resolutions, data format (bit depth), etc. These differences can have a small impact on the result of the segmentation algorithm. As suggested by Chen et. al. [22], it might therefore be beneficial to first apply signal normalization to reduce the A-scan differences between different instruments. In the present case, it is more likely that the difference between Heidelberg and Cirrus OCT is to some extent caused by the use of averaging with Heidelberg—in the present study, averaging of 100 A-scans was applied. The averaging mechanism will lead to an enhancement of small, consistent signals from multiple scans, and this may lead to an increase in the measured thickness, as the lower limit of the outermost RPE is positioned deep with weak signals on the posterior border [23]. In addition, if the B-scans cannot be perfectly registered/aligned, one will obtain a small blurring effect that will also lead to an apparent greater thickness. For the thickness of the RPE_OS_complex_, a similar effect may exist both for the IS/OS line and the posterior border of the RPE.segmentation line. We found a thickness of the RPE_OS_complex_ to be approximately 8 μm larger than that of the published data from Wang et al. [24] based on UHR-OCT and another segmentation algorithm. A previous study of healthy subjects using TD OCT and a segmentation algorithm similar to the one used in the present study gave comparable results to our study [25]. It should be noted that the difference in both retinal thickness and RPE_OS_complex_ is below the axial resolution of the devices (5 μm for Cirrus SD OCT).

The eyes included in this study were fellow eyes in patients with epiretinal membrane on the other eye. The included eyes had normal visual acuity and were evaluated clinically to be without ocular pathology, and the mean central subfield thickness (CSF) was comparable to results reported for he Heidelberg Spectralis and Cirrus HD [4, 7, 9]. A subclinical increase cannot be totally ruled out, but is assumed to be very small.

A weakness of the present study is the subjective assessment of scan positions, which cannot be avoided for different instruments and the many differences in scanning protocols. Despite these shortcomings, the comparison of the Cirrus to the Heidelberg Spectralis showed a high degree of agreement.

In conclusion, the differences in retinal thickness calculated by spectral OCT are primarily due to differences in software segmentation definitions, and not to the instruments. However, the thickness was consistently greater with the Heidelberg, the mean difference being 1.85 μm, corresponding to 1 %. For the RPE_OS_complex_, with the intraretinal segmentation line at the level of the inner and outer photoreceptor segments, the difference was 3.32 μm, corresponding to 5 %. In clinical use, the differences are negligible if the same software is used for the Cirrus HD OCT and the Heidelberg Spectralis.
